# Tick-borne encephalitis epidemiology and surveillance in Poland, and comparison with selected European countries before and during the COVID-19 pandemic, 2008 to 2020

**DOI:** 10.2807/1560-7917.ES.2023.28.18.2200452

**Published:** 2023-05-04

**Authors:** Iwona Paradowska-Stankiewicz, Katarzyna Pancer, Anna Poznańska, Martyna Hordowicz, Maria Skibicka, Marek Słowiński, Gerard Motak, Bogdan Falkiewicz

**Affiliations:** 1 Department of Epidemiology of Infectious Diseases and Surveillance, National Institute of Public Health NIH – National Research Institute, Warsaw, Poland; 2 Laboratory BSL3 and Virology Department; National Institute of Public Health NIH – National Research Institute, Warsaw, Poland; 3 Department of Population Health Monitoring and Analysis, National Institute of Public Health NIH – National Research Institute, Warsaw, Poland; 4 General Psychiatry Unit III, Dr Barbara Borzym’s Independent Public Regional Psychiatric Health Care Centre, Radom, Poland; 5 Pfizer Polska Sp. z o.o., Vaccines Poland, Warsaw, Poland; 6 IQVIA Commercial Consulting sp. z o.o., Warsaw, Poland

**Keywords:** tick-borne encephalitis, virus, central nervous system, TBE, TBEV, NIPH NIH-NRI, epidemiological surveillance, viral meningoencephalitis

## Abstract

**Background:**

Tick-borne encephalitis (TBE) is the most common viral central nervous system (CNS) infection in Poland. Previous research suggests that its incidence was underestimated in the pre-pandemic period. The COVID-19 pandemic caused a considerable burden on surveillance systems, which could further impact reporting.

**Aim:**

We aimed to assess the completeness of reporting of TBE in the years 2008 to 2020 and explore the potential impact of the COVID-19 pandemic on reporting to the epidemiological surveillance system, compared with hospitalisations for TBEV and other viral neuro-infections.

**Methods:**

We compared the Polish epidemiology of TBE and other viral infections of the CNS from national surveillance reports with data on hospitalisations from 2008 to 2020 and data from selected European countries.

**Results:**

Between 2008 and 2020, 3,016 TBE cases were reported to surveillance compared with 3,620 hospitalisations. There was an increasing trend in hospitalisations, while surveillance data demonstrated the opposite, with the largest discrepancy observed in the first pandemic year (354 hospitalisations vs 159 cases reported to surveillance). Serological testing for TBE was used more in the known endemic region of north-eastern Poland and less in non-endemic areas. Other European countries reported higher TBE case numbers and an increase during the COVID-19 pandemic, whereas Poland observed an opposite trend.

**Conclusion:**

The sensitivity of TBE surveillance in Poland requires improvement. There are considerable regional differences. Regions that test for TBE intensively report most cases. Policymakers should be made aware of the value of quality epidemiological data for planning prophylactic measures in risk areas.

**Key public health message**

**What did you want to address in this study?**

Understanding the local epidemiology of vaccine-preventable diseases is critical for establishing prophylactic measures. Tick-borne encephalitis (TBE) cases should be reported to the national surveillance system. We compared the reported case numbers with the number of patients hospitalised for TBE to see how complete the reporting is and also compared them with other European countries; a large difference might indicate that cases in Poland are underestimated.

**What have we learnt from this study?**

Our findings suggest that the number of cases of TBE in Poland are underestimated, because more patients were hospitalised for TBE than recoded by the surveillance. Suboptimal use of laboratory diagnostics to identify of TBE cases, was one of the probable causes.

**What are the implications of your findings for public health?**

TBE is Poland's most common cause of viral encephalitis; therefore, efficient and high-quality monitoring of its occurrence is essential for planning adequate prophylactic measures. We should raise awareness among hospital managers and the National Health Fund to expand diagnostics for central nervous system infections, including TBE.

## Introduction

The COVID-19 pandemic posed several challenges to the healthcare system, but the implications for epidemiological surveillance systems might be overlooked. An increased workload for healthcare workers may interrupt the continuity of reporting and monitoring of the epidemiological situation of other diseases as more priority is given to reducing the impact of the pandemic on the community.

Tick-borne encephalitis (TBE) is Poland’s most common cause of viral encephalitis among central nervous system infections [1]. The course of the disease can make diagnosis difficult, given the non-specific symptoms that resemble other aseptic central nervous system (CNS) infections [2-5]. The first phase of the disease lasts 2–4 days and is characterised by influenza-like symptoms. The symptoms of the second phase, estimated to affect 20–30% of cases, are specific enough to suggest a disease of the CNS, urging the physician to initiate the diagnostic process. Currently, no available drug or therapeutic solution can effectively treat TBE [2,6]. Available preventive measures against TBE involve vaccination and protection against tick bites [7]. Therefore, confirmation of TBE diagnosis is critical to understand the risk of the disease in different regions, as is the setting up of adequate preventive measures to avoid complications that might lead to permanent impairment [1]. 

According to the case definition from the European Centre for Disease Prevention and Control (ECDC), which is also used in Poland, the diagnosis of TBE is based on clinical criteria and laboratory tests of blood or cerebrospinal fluid [8,9]. Due to the invasiveness of the procedure, lumbar puncture is less commonly performed. Often, TBE is confirmed through serological testing and detection of TBEV-specific IgM and IgG antibodies from serum samples [10]. While PCR-based tests allow for the multiplexed detection of TBEV nucleic acid, these are not used in routine clinical practice [9]. Scientific research on the spread of the disease and the infectiveness of ticks also uses PCR-based tests. Nonetheless, use of diagnostic testing in Poland appears to be insufficient, most often for pragmatic (no impact on clinical management) and financial reasons (confirmation of TBEV infection does not change the valuation of the hospitalisation cost by the National Health Fund) [11]. Therefore, hospital managers and physicians perceive that identifying the aetiological factor in every case is unnecessary. 

Infections with TBEV have been increasing throughout Europe, and in 2012, TBE became a notifiable disease in the European Union [12,13]. Currently, reporting cases of TBE to the National Institute of Public Health National Institute of Hygiene – National Research Institute (NIPH NIH-NRI) is obligatory in Poland according to the Act of 5 December 2008 on preventing and combating infections and infectious diseases in humans [14]. Contrary to what is observed throughout Europe, Polish epidemiological data collected by the NIPH NIH-NRI indicate a decreasing trend for TBEV infections. Other researchers have previously raised issues with TBE reporting in Poland. Scientists from the University of Białystok pointed out the incompleteness of data from the epidemiological surveillance system based on comparing surveillance data with the number of hospitalisations at a local infectious disease clinic [15]. Moreover, Stefanoff et al. demonstrated outbreaks of TBE in Polish territory, which were not included in the epidemiological surveillance system reports [16]. 

As TBEV is not the only cause of aseptic CNS infection, it is necessary to include other aetiological factors in the differential diagnosis, particularly other viruses. An analysis of the occurrence of TBEV in Poland in 2018 highlighted the underuse of aetiological testing by emphasising the high share of diagnoses of neuro-infection without aetiological confirmation [17]. Annual Polish epidemiological reports demonstrate that around 70% of meningitis and encephalitis cases are being reported without determining the causative agent, among which there are potentially unidentified TBE cases [18]. For these reasons, TBE reporting might reflect the sensitivity of the surveillance system as a whole.

This study had several aims. Based on the available literature, we hypothesised that the sensitivity of the TBE surveillance system in Poland is low. Our main aims were to confirm such a hypothesis and to assess the impact of the COVID-19 pandemic on reporting TBE cases to the epidemiological surveillance system compared with data from the Nationwide General Hospital Morbidity Study (NGHMS), which collects data about hospitalisations for TBEV and other viral neuro-infections. These datasets differ in their data collection methodologies. Comparing them allows us to assess the sensitivity of the surveillance system and the possibility of underestimating the number of reported TBE cases. We further aimed to compare these datasets with selected European countries representing different surveillance types. Additional objectives of this research were to identify possible sources of discrepancies between the number of cases reported in distinct databases. We also compared the number of TBEV tests performed in Poland in each voivodeship (16 regions in Poland) with regional epidemiological data.

## Methods

### Surveillance system and data acquisition methods

The epidemiological surveillance system in Poland performs data collection and analysis of infectious diseases epidemiology and associated risks. Epidemiological surveillance in Poland is based on electronically collected, verified, analysed, and interpreted data on infections and infectious diseases [14]. Physicians are obliged to report communicable disease cases to the local sanitary inspectorates, who check the reports for completeness and transfer the aggregated data to the regional sanitary inspectorates, together with case-based questionnaires. Regional sanitary inspectorates transfer that data to the NIPH NIH-NRI. After verification and analysis, the NIPH NIH-NRI publishes national-level data in periodic, biweekly reports. They contain aggregated data, while the annual report entitled *Bulletin of infectious diseases and intoxications in Poland* also contains regional-level data [18]. In addition, NIPH NIH-NRI is the national unit responsible for submitting specific data to European institutions such as the ECDC or World Health Organization (WHO).

### Case definition and clinical characteristics

This study used the TBE definition according to the NIPH NIH-NRI 2020 edition of *Definitions of infectious disease cases for epidemiological surveillance purposes* [9], which is synonymous with the ECDC definition [8]. Confirmation of a TBE case in Poland is possible when both the clinical and laboratory criteria of a confirmed case are met. The clinical features of TBE are signs of inflammation of the central nervous system, which include meningitis, meningoencephalitis, encephalomyelitis, or encephalo-radiculitis [9]. The laboratory criteria of a confirmed case comprise (i) simultaneous detection of IgM and IgG antibodies characteristic of TBEV infection in the blood, (ii) detection of TBEV-specific IgM antibodies in cerebrospinal fluid, (iii) detection of seroconversion in two serum samples or a fourfold increase in TBEV-specific antibody titres or (iv) detection of TBE virus genetic material or isolation of TBEV in clinical specimens [9]. For a probable case, the detection of sole IgM in a single serum sample is sufficient [9].

### Data sources and statistical analysis

NIPH NIH-NRI reports were the primary source of data in this study. Data on hospitalisations from the NGHMS, collected by NIPH NIH-NRI as directed by the Ministry of Health, were used for comparative analysis. The survey and the epidemiological surveillance system were based on the ICD-10 classification of diseases [19]. We also used data from European countries collected by ECDC. The notification rates for TBE are given per 100,000 inhabitants. The period analysed for European data ranged from 2015 to 2020, where available [20,21]. However, it should be noted that the definition of a confirmed case of TBE is not the same in all countries. 

The NIPH NIH-NRI data included national and regional epidemiological data of encephalitis or meningitis cases reported to the surveillance system [17,18]. The analysed period was 2008 to 2020. Data until 2019 refer to confirmed cases and for 2020, the analysed data refer to confirmed and probable cases. We used the probable cases as a proxy for the maximum number of all cases, temporarily awaiting confirmation.

The NIPH NIH-NRI organised the NGHMS as directed by the Ministry of Health. The data included in the analysis refer to the number of hospitalisations for tick-borne encephalitis (A84), viral encephalitis (A86), and meningitis (A87) for the years 2008 to 2020. The research from which the data on hospitalisations are derived is part of the survey ‘*Hospitalisation*’ covered by the Programme of Public Statistical Research. It collects unit data on all hospitalised cases in Poland in the scope compliant with the general hospital statistical card. Data on hospitalisations are collected regardless of a patient's place of residence, citizenship or insurance status. The transferred data are anonymous; they do not include personal details or other identifiers such as the patient’s insurance number. The obligation to provide data for the survey applies to entities performing medical activity subordinate to the Ministry of Health, Ministry of National Defence, and Ministry of Interior and Administration, except for facilities included in the Council of Ministers' ordinance. It lists inpatient hospices and psychiatric hospitals, among others.

We conducted a comparative analysis to assess the consistency of national epidemiological data from NIPH NIH-NRI with data from the NGHMS; we also analysed differences in reporting between regions. The collected data were analysed using a spreadsheet. Bar graphs, line graphs, and cartograms were used for data visualisation, and MS Excel version 2019 was used for data analysis.

## Results

### Comparison between data from the epidemiological surveillance system and the NGHMS in Poland

**Figure 1:**
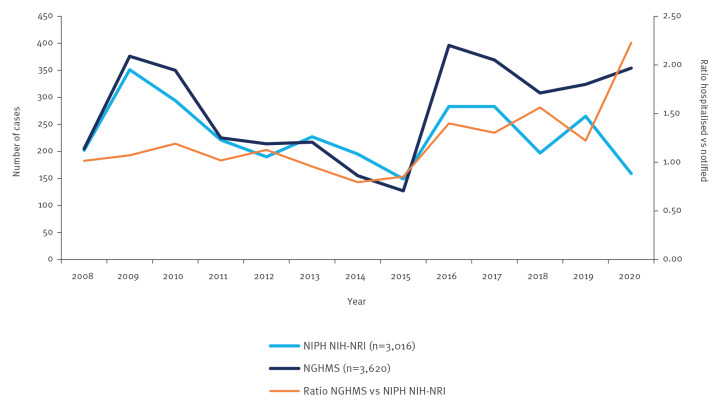
Hospitalisations for tick-borne viral encephalitis vs notifications to NIPH NIH-NRI, Poland, 2008–2020

**Figure 2:**
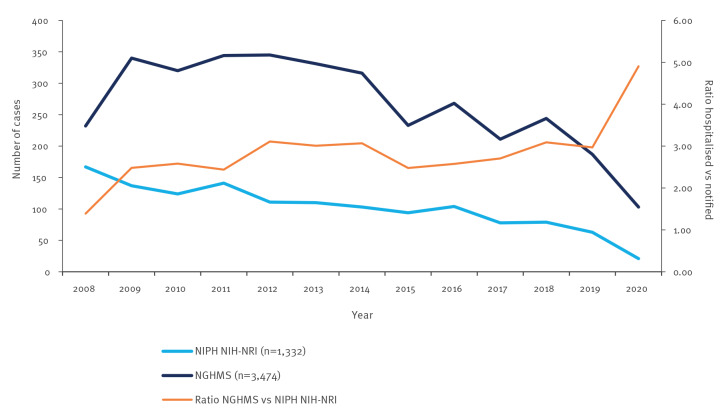
Hospitalisations for unspecified viral encephalitis vs notifications to NIPH NIH-NRI, Poland, 2008–2020

Between 2008 and 2020, respectively 3,016 and 3,620 cases of TBE (A84) were reported to the surveillance system and hospitalisations database. The case numbers reported for unspecified viral meningitis (A86) were 1,332 and 3,474, respectively. Figure 1 shows an increasing number of TBE hospitalisations reported in the NGHMS across the analysed period, while the NIPH NIH-NRI reports demonstrate the opposite. Figure 2 illustrates the case numbers for unspecified viral meningitis for both databases.

NGHMS: Nationwide General Hospital Morbidity Study; NIPH NIH-NRI: National Institute of Public Health NIH – National Research Institute.

Cases registered as per IDC10 code A84 for viral encephalitis: tick-borne.

NGHMS: Nationwide General Hospital Morbidity Study; NIPH NIH-NRI: National Institute of Public Health NIH – National Research Institute.

Cases registered as per IDC10 code A86 for viral encephalitis: unspecified.

Between 2013 and 2014, NIPH NIH-NRI changed the definition of a confirmed TBE case by [22,23]. Since 2014, it has been possible to classify a confirmed case based on detection of TBEV RNA in blood by RT-PCR; however, this method is only used to a limited extent. In addition, the neutralisation test criterion was removed from the laboratory criteria in 2014 as it is very rarely used in practice because there is no reference laboratory for arboviral infections in Poland and the associated costs are high [23,24]. Cases of TBE and unspecified viral encephalitis ratios between numbers reported by both databases, are shown in Figures 1 and 2.

At the beginning of the study period 2008 to 2012, differences between the number of TBE cases reported by NIPH NIH-NRI and NGHMS were small. In the following years (2013–2015), the number of cases recorded by NGHMS was smaller than in the NIPH NIH-NRI reports. This trend was reversed in 2016. The difference in the number of TBE cases reported to the NGHMS and NIPH NIH-NRI became considerable (mean difference: five cases in the period 2008–2015 vs 112.8 cases in 2016–2020). According to NGHMS data, the annual number of TBE-related hospitalisations increased from 127 in 2015 to 396 cases in 2016 (more than threefold). In the NIPH NIH-NRI reports the number of cases increased by 90% (149 in 2015 vs 283 in 2016). The difference between the systems reached 113 cases in 2016, which corresponds to 40% of the total cases reported to NIPH NIH-NRI.

**Figure 3:**
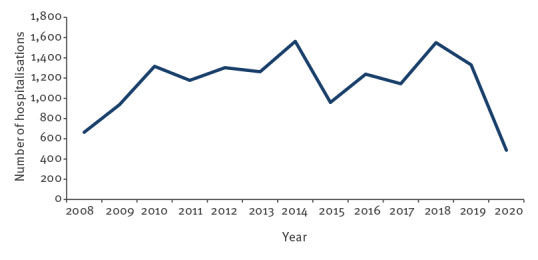
Hospitalisations caused by viral meningitis according to the NGHMS, Poland, 2008–2020 (n = 14,933)

Interestingly, in the period 2016 to 2020, there was a substantial difference in the number of hospitalisations for TBE (A84) between NIPH NIH-NRI and NGHMS (Figure 1). In those 5 years, the cumulative difference reached 564 cases, more than reported to the surveillance system yearly. A similar phenomenon was observed for unspecified viral encephalitis, with a difference of 371 cases reported between 2018 and 2020 (Figure 2). As shown in Figure 3, there has also been a notable decrease in viral meningitis cases (A87) reported to the NGHMS since 2018. In 2018, 1,550 cases were recorded in the NGHMS, and in 2020, the number of recorded cases decreased by almost a thousand (a 69% decrease) [17].

NGHMS: Nationwide General Hospital Morbidity Study.

Cases registered as per ICD-10 code A87 for viral meningitis.

During the first pandemic year, 2020, the discrepancies between the TBE cases reported to the analysed databases were the largest in the whole study period. The NGHMS recorded 354 hospitalisations for TBE. Compared with the beginning of the observation period, this was an increase by 149 cases (73%). The number in 2020 was 11% lower than the historical maximum recorded in 2016 and 280% higher than the historical minimum recorded in 2015. At the same time, unspecified viral encephalitis cases in 2020 decreased to a historical minimum. For more details please refer to Figures 1 and 2.

The number of hospitalised TBE patients in 2020 reported in NGHMS was 2.23 times higher (by 123%) than the number reported to the national surveillance (NIPH NIH-NRI). The reporting rate in the pre-pandemic year 2019 was much higher: 82% of hospitalised cases were also reported to the NIPH NIH-NRI. In contrast, at the beginning of the analysed period in 2008, the difference between the cases reported to NIPH NIH-NRI and NGHMS was negligible, with an excess of only 1%. There was, however, a period (2013–2015) during which the number of cases reported to NIPH NIH-NRI was higher than recorded in NGHMS. 

The discrepancies in reporting during the first year of the pandemic were also observed for other viral neuro-infections. In 2019, NGHMS recorded 2.97 times the number of unspecified viral meningitis cases than NIPH NIH-NRI, and this difference increased to nearly five-fold in 2020 (103 vs 21 cases). In contrast, the number of cases reported by NGHMS in 2008 was only 1.39 times the number reported to NIPH NIH-NRI. 

**Figure 4:**
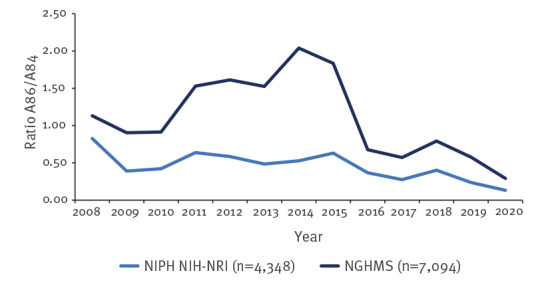
Ratio of unspecified vs tick-borne viral encephalitis cases for NIPH NIH-NRI and NGHMS data, Poland, 2008–2020

The ratio of reported cases of unspecified viral encephalitis vs TBE in the NIPH NIH-NRI database was consistently below the rate in NGHMS, suggesting a different proportion of cases reported to NIPH NIH-NRI from both groups of encephalitis (Figure 4). At the beginning of the study period, the ratio was 1.13 for NGHMS and 0.83 for NIPH NIH-NRI, a historical maximum for the latter. In the NGHMS data, the historical maximum was in 2014, with a ratio of 2.04. The ratio decreased from 2018 to 2020 and reached 0.29 for NGHMS and 0.13 for NIPH NIH-NRI, the historical minimum for both databases; this represents a 50% decrease. For more details about the number of cases reported to the surveillance and the hospitalizations databases by ICD_10 codes, please refer to Supplementary Table S1.

NGHMS: Nationwide General Hospital Morbidity Study; NIPH NIH-NRI: National Institute of Public Health NIH – National Research Institute.

Cases registered as per IDC10 code A84 for viral encephalitis: tick-borne and code A86 for encephalitis cases: unspecified.

### Tick-borne encephalitis in selected European countries

We compared the Polish data with data from European surveillance systems, based on geographical proximity (e.g. Latvia, Germany, Lithuania, Slovakia) and known endemic regions for TBE (Austria, Sweden, Estonia). For this purpose, we used data from the ECDC *Annual Epidemiological Report* for the period 2015 to 2020 (Table) covering only confirmed cases of TBE (A84) [20,21].

**Table:** Confirmed tick-borne encephalitis cases, selected countries in the European Union, 2015–2020 (n = 12,233)

Country	2015		2016		2017		2018		2019		2020	
	n	per 100,000	n	per 100,000	n	per 100,000	N	per 100,000	n	per 100,000	n	per 100,000
Austria	79	0.9	96	1.1	123	1.4	170	1.9	106	1.2	250	2.8
Estonia	115	8.7	80	6.1	84	6.4	85	6.4	82	6.2	70	5.3
Finland	68	1.2	61	1.1	82	1.5	79	1.4	69	1.3	91	1.6
Germany	218	0.3	353	0.4	486	0.6	583	0.7	445	0.5	705	0.8
Latvia	141	7.1	91	4.6	178	9.1	100	5.2	118	6.1	149	7.8
Lithuania	336	11.5	633	21.9	474	16.6	384	13.7	711	25.4	679	24.3
Poland^a^	115	0.3	211	0.6	196	0.5	148	0.4	197	0.5	114	0.3
Slovakia	80	1.5	169	3.1	75	1.4	156	2.9	161	3	185	3.4
Sweden	268	2.7	238	2.4	365	3.7	359	3.5	355	3.5	267	2.6

^a^ Only confirmed cases are included, so data with some difference to the numbers reported previously.

Source: [20,21].

Between 2015 and 2020, most selected countries observed an increase in TBE cases. In 2020, the first pandemic year, the numbers in most countries were stable or increased compared with 2019, except Estonia, Sweden, Lithuania and Poland. Nonetheless, in 2019 and 2020, TBE incidence in Lithuania peaked, reaching 25.4 and 24.3, respectively, compared with the period 2015 to 2018, with an average of 15.9. In 2020, TBE cases increased compared with 2019 by 136%, 58%, and 32% in Austria, Germany, and Finland, respectively, whereas in Poland, reported TBE cases decreased by 42%. In Austria, as many as 120% more cases were reported in 2020 than the 2015 to 2019 period average; in Germany, the TBE case number in 2020 increased by 70%, compared with the average from 2015 to 2019. In Poland, the incidence rate per 100,000 population in 2020 was 0.3, the lowest value among the selected countries in that year. The second-lowest incidence was recorded in Germany with 0.8 cases of TBE per 100,000 population, but this was more than two times higher than in Poland. The highest rate was recorded in the Baltic states (Table) [21,25].

### Case numbers and serological tests for tick-borne encephalitis in different regions of Poland

**Figure 5:**
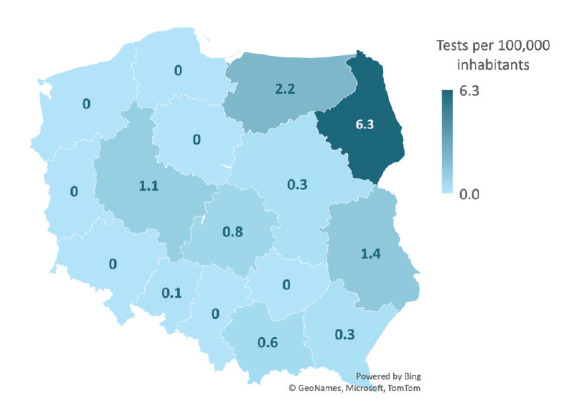
Hospitalisations for tick-borne encephalitis with diagnostic procedure F84 (anti-TBEV IgG), in relation to the number of inhabitants, NGHMS data, Poland, 2020 (n = 297)

NGHMS: Nationwide General Hospital Morbidity Study; TBEV: tick-borne encephalitis.

**Figure 6:**
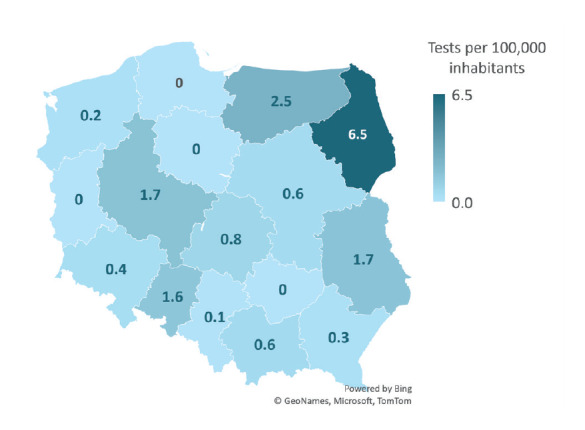
Hospitalisations for tick-borne encephalitis with diagnostic procedure F85 (anti-TBEV IgM), in relation to the number of inhabitants, NGHMS data, Poland, 2020 (n = 379)

NGHMS: Nationwide General Hospital Morbidity Study; TBEV: tick-borne encephalitis.

We analysed the number of TBE diagnostic tests performed to investigate whether the regional variation in testing corresponded with the number of reported cases. The endemicity of TBE in some regions might be related to a higher number of diagnostic tests performed. Two procedures were included in the analysis – F84 and F85, detecting, respectively, TBEV-specific IgG antibodies and IgM antibodies. The data demonstrated a link between the number of tests performed in a region and reported TBE cases (Figures 5 and 6). For further reference on the geographical distribution of TBE and other viral CNS infections, we provide that information in the Supplementary Figures and Tables S2–S4. 

The number of F84 procedures performed in 2020 was highest in Podlaskie voivodeship, which is known to be a hyperendemic region for TBE with an annual incidence > 5 per 100,000 population; the second-highest number of tests was performed in Wielkopolska, while Warmian-Masurian Voivodeship ranked third. From 2017 to 2019, the total number of tests performed increased, with an average annual growth rate of 19%. From 2019 to 2020 (COVID-19 pandemic period), there was a marked reduction in the number of F84 tests performed, from 603 procedures in 2019 to 297 in 2020 (a 51% decrease). The voivodeships with the highest number of F85 procedures performed were Podlaskie, Łódź Voivodeship and Warmian-Masurian Voivodeship. There was also an increase in the number of tests performed from 2017 to 2019, with an average annual growth rate of 19%. In contrast to the F84 procedure, there was a drastic decrease (by 42%) in 2020. A similar regional distribution may be found in the case of IgM antibody testing. Again, Podlaskie voivodeship performed most tests (Figure 6). Four of 16 voivodeships used 0 tests per 100,000 inhabitants, whereas only five, including Podlaskie region, conducted more than one test per 100,000 habitants.

## Discussion

The data from the national epidemiological surveillance – NIPH NIH-NRI – should correspond overall with the data on hospitalisations from the NGHMS. Nonetheless, our analysis demonstrated major discrepancies between the two and that the number of TBE cases reported to the surveillance system in Poland is an underestimate. Our findings align with other research in both recent and older publications [1,15,16,26]. An apparent decline in the number of reported cases occurred in 2020, coinciding with the start of the COVID-19 pandemic and the associated increased burden on the surveillance system and health services, which negatively impacted the quality of epidemiological surveillance system data. That was evidenced by comparing NIPH NIH-NRI data against the NGHMS reports on hospitalisations which did not show the same decline in TBE diagnoses. The discrepancy between NIPH NIH-NRI and NGHMS data was also noticeable in previous years, although less pronounced than in 2020. It is therefore reasonable to assume that the the COVID-19 pandemic negatively impacted the efficiency of TBE case reporting from Polish hospitals to the Polish surveillance system. Therefore, the true incidence of TBE in 2020 might be reflected more accurately in the NGHMS database, which aims to collect direct data on hospitalisations; one plausible explanation is that the transfer of data to the NGHMS database is less time-consuming for the reporting physician than providing a detailed report to the case-based surveillance system.

Variation in TBE incidence across different voivodeships may be related to the performance and sensitivity of epidemiological surveillance within Poland. The incidence of TBE in 2020 in Podlaskie Voivodeship was 6.63 per 100,000 inhabitants compared with 2.11 in the Wamian-Masurian and 0.35 in Lesser Poland Voivodeship [27]. Podlaskie Voivodeship is known as Poland’s most endemic region also for unspecified viral encephalitis [1,15,16], and it has been a well-established endemic area of TBEV for decades [1,16]. Our study demonstrates that serology testing for TBE infection is more often used there, which may have further strengthened the epidemiological surveillance of TBE in this voivodeship, making it more effective than in the rest of Poland. Other authors have described similar findings in previous years. Stefanoff et al. suggested that considerable variation in its sensitivity and specificity exist between the voivodeships. The detection rate in Poland for TBE has been low for at least 12 years [16]. The authors also developed a mathematical and statistical predictive model which indicated that there might be more TBE cases in most regions than reported [26]. Studies by Sulik et al. and Paradowska-Stankiewicz and Zbrzeźniak have also demonstrated that the reported number of TBE cases does not reflect the actual burden of the disease [1,15]. They suggested that it is likely to be high also in some voivodeships that are not currently considered endemic regions for TBEV. 

The regional variation in the number of tests performed suggests a lack of awareness of how individual aetiological testing contributes to the overall quality of epidemiological data. If this is the case, there is a need to educate physicians, laboratory diagnosticians, and other healthcare professionals about the role of proper identification of the aetiological agent in defining TBE risk areas and designing adequate measures for TBE prevention such as vaccination programmes for populations living in highly endemic regions or with the highest exposure to tick bites. Another explanation for the low use of TBE tests in some areas may be a belief, based on available surveillance data, that the likelihood of TBEV infection is low or has decreased. That would result in under-reporting and low case numbers, which might further discourage physicians from performing confirmatory tests for TBE. The Polish surveillance systems’ sensitivity for neuro-infections might be improved by encouraging the use of laboratory tests that confirm the aetiology of the viral CNS infections.

Comparing Polish TBE incidence rates with other European countries indicates that the number of TBEV infections in Poland is probably not reflected accurately in epidemiological registers. Combined with the low use of TBE tests in most voivodeships, it is highly probable that some cases are reported in the category of CNS viral infections ‘other and unspecified’. Nonetheless, a direct comparison of incidence rates alone has some limitations, as it does not consider the difference in case definitions across European countries. The Polish case definition for confirmed TBE involves both laboratory criteria and symptoms of CNS involvement. In contrast, the Finnish and Norwegian case definitions require only laboratory findings without specified clinical presentation. Some countries also report patients with isolated febrile symptoms and no evidence of neurological involvement if laboratory tests confirm TBEV infection (Austria, Germany and Switzerland) [25]. These discrepancies have to be considered when interpreting international surveillance data. 

In 2019, according to the official national data, 87,917 people were newly vaccinated against TBEV in Poland, corresponding to roughly 0.22% of the country’s population [28]. This is much less than estimated by some studies, where vaccination coverage was calculated based on participants’ testimonies in a population sample [29]. Only 5,541 (6.3%) of these vaccinees resided in Podlaskie voivodeship highly endemic for TBE [28]. It is, therefore, vital to continue efforts to increase awareness of the value of vaccination against TBE in known endemic areas. In addition, improving TBE surveillance would help identify other regions with a high risk of the disease, where vaccination should be encouraged [1]. Increasing the surveillance system's sensitivity for TBE might be acheived by introducing systemic changes connected with a higher valuation of procedures, including diagnostics of viral CNS inflammations by testing the aetiological agent. Low quality of TBE data and lack of widely used prophylaxis may increase the disease burden and its sequelae [1,15].

## Conclusions

The sensitivity of the Polish epidemiological surveillance system for TBE still needs to improve. Deficiencies lead to under-reporting of TBE cases that meet the ECDC definition for TBE and to low quality of regional and national epidemiological data. The main reason for this is probably a suboptimal use of laboratory diagnostics allowing identification of the aetiological agent in patients with viral infection of the CNS. Activities should be implemented to raise the awareness of hospital managers and the National Health Fund of the need to expand the diagnostics of neuro-infections to include tests for TBEV, particularly outside known endemic areas.

## Supplementary Data

369000 Supplement
